# Ecosystem Function in Appalachian Headwater Streams during an Active Invasion by the Hemlock Woolly Adelgid

**DOI:** 10.1371/journal.pone.0061171

**Published:** 2013-04-22

**Authors:** Robert M. Northington, Jackson R. Webster, Ernest F. Benfield, Beth M. Cheever, Barbara R. Niederlehner

**Affiliations:** Virginia Polytechnic Institute and State University, Department of Biological Sciences, Blacksburg, Virginia, United States of America; University of Illinois Urbana-Champaign/Energy Biosciences Institute, United States of America

## Abstract

Forested ecosystems in the southeastern United States are currently undergoing an invasion by the hemlock woolly adelgid (HWA). Previous studies in this area have shown changes to forest structure, decreases in canopy cover, increases in organic matter, and changes to nutrient cycling on the forest floor and soil. Here, we were interested in how the effects of canopy loss and nutrient leakage from terrestrial areas would translate into functional changes in streams draining affected watersheds. We addressed these questions in HWA-infested watersheds at the Coweeta Hydrologic Laboratory in North Carolina. Specifically, we measured stream metabolism (gross primary production and ecosystem respiration) and nitrogen uptake from 2008 to 2011 in five streams across the Coweeta basin. Over the course of our study, we found no change to in-stream nutrient concentrations. While canopy cover decreased annually in these watersheds, this change in light penetration did not translate to higher rates of in-stream primary production during the summer months of our study. We found a trend towards greater heterotrophy within our watersheds, where in-stream respiration accounted for a much larger component of net ecosystem production than GPP. Additionally, increases in rhododendron cover may counteract changes in light and nutrient availability that occurred with hemlock loss. The variability in our metabolic and uptake parameters suggests an actively-infested ecosystem in transition between steady states.

## Introduction

Biological invasions have the potential to greatly affect ecosystem structure and processes. Alterations in compositional structure of ecosystems are one of the most direct effects of invaders, while changes in biogeochemical cycles or productivity are a secondary consequence [1], [2]. Significant modifications to carbon [3] and nitrogen [4], [5] inputs along with shifts in forest community composition [6], [7] have been noted throughout the extent of the infestations by both fungal [8] and insect invaders [9], [10], [11], [12]. Previous work in Coweeta [13] noted significant losses of riparian canopy cover and subsequent increases in light intensities and stream temperatures, all attributable to the influence of the hemlock woolly adelgid (HWA, *Adelges tsugae*).

Currently, forests spanning the eastern United States are being invaded by the HWA [14], which are limited by water availability and temperature [15]. Even so, their range may continue to expand with increases in annual warming [16]. HWA was first noted in southeastern forests in 2003 [17]. Within 5 years, there was 33% tree mortality [13], and currently there is a near total loss of hemlock trees [18]. The most direct effect of HWA appears to be increased canopy openness [13], [19], which is allowing for previously suppressed species such as oaks, hardwoods, and rhododendron (*Rhododendron* maximum) to become more abundant [6], [7]. Thus, an overstory becoming more dominated by deciduous trees will allow more light to reach both the forest floor and stream ecosystems during times of leaf-off [6], [13].

Concurrent with an increase in canopy openness will be inputs of large hemlock wood and needles to both the forest floor [4] and streams [13]. In-stream concentrations of nitrate have increased significantly in some watersheds with death of hemlocks resulting from the leaching of nitrogen [5], [20], in addition to increased annual discharge brought about due to a shift to a more deciduous watershed [21]. Thus, increases in light and nutrient availability may increase in-stream primary production [22]. Over the long term, nitrogen may eventually be retained in the forest floor due to immobilization of the nitrate by heterotrophic microbes colonizing the newly available litter resources [3], [4], [23].

Forest disturbance has been shown to have a significant impact on ecosystem processes in streams. As noted earlier, increases in canopy openness allows for greater light availability to streams in watersheds affected by HWA [13], [19]. A significant relationship between canopy openness and primary production has been noted in a variety of stream ecosystems spanning the globe [24], [25]. Earlier studies in Coweeta [26] and British Columbia [27] demonstrated significant increases to periphyton biomass in clearcut watersheds, mostly due to increases in light penetration.

Measures of watershed-scale processes, such as nutrient uptake and stream metabolism, are valuable tools to assess the role of invasive species as a disturbance within an ecosystem. Biogeochemical cycling in streams has been a key component of many ecosystem studies, as it integrates changes to nutrients over large scales of time and space into smaller, more measurable units of study [28], [29]. Additionally, relating carbon dynamics (fixation and mineralization) with nutrient demand helps to better assess the energetics of an ecosystem [30], [31], [32], especially one that is going through an active disturbance. Therefore, streams are ideal for measuring ecosystem response to a wide variety of disturbances, especially given the current ease of capturing changes to nutrients and metabolism over a range of time scales [33], [34]. In this study, we assessed the indirect effect of a current HWA infestation on ecosystem function in streams within watersheds where riparian hemlock is abundant. We hypothesized that changes in metabolism and nutrient uptake should occur because of a loss of riparian hemlock canopy. As the canopy around streams becomes more open, we predict that in-stream primary production should increase. Additionally, greater leaching of nutrients from hemlock-dominated watersheds should increase nutrient uptake by autotrophs and stimulate primary production.

## Materials and Methods

### Study Sites

This study was performed at Coweeta Hydrologic Laboratory in southwestern North Carolina (35°03′35″N, 83°25′51″W) as part of long-term research of the effects of HWA on forest canopy structure and ecosystem function in this region (e.g. [13]).

Previous work on ecosystem effects of HWA was conducted using 9 sites within the larger Coweeta Creek watershed; here, we chose a subset of five lower order streams (1^st^–2^nd^) used in a previous study in Coweeta [13]. The streams used in the present study are physically similar (Table 1) and have low background concentrations of important nutrients, such as inorganic nitrogen (Table 2). Hemlock basal area contribution to vegetated riparian corridors surrounding the streams varied from 28.4% (Hugh White Creek, Lower) to a high of 41.6% in Mill Branch [13]. We did not use reference non-hemlock sites due to the prevalence of this species in forests of this region.

**Table 1:** Stream characteristics. Physical measures of the 5 study streams in Coweeta during July 2008–2011.

	Temperature (°C)		Discharge (L s^−1^)		Width (cm)		Depth (cm)	
Stream	Range	Mean (SE)	Range	Mean (SE)	Range	Mean (SE)	Range	Mean (SE)
Cunningham (Cunn)	16.3–17.4	17.0 (0.2)	2.7–9.6	7.4 (1.6)	174.3–199.2	190.2 (5.6)	3.5–5.3	4.3 (0.4)
Hugh White- Lower (HWCL)	17.5–19.8	18.2 (0.6)	1.2–7.3	5.0 (1.4)	172.7–227.8	194.1 (11.9)	1.8–4.2	2.9 (0.5)
Hugh White- Upper (HWCU)	16.6–18.1	17.3 (0.3)	1.6–6.6	4.3 (1.0)	220.1–240.0	229.4 (5.1)	2.0–3.1	2.6 (0.3)
Mill	16.2–17.2	16.8 (0.2)	1.3–6.7	4.5 (1.2)	185.4–227.9	206.8 (9.8)	1.6–3.4	2.6 (0.4)
Reynolds (Reyn)	14.1–16.5	14.9 (0.5)	1.2–5.4	3.4 (0.9)	182.1–302.6	223.3 (27.2)	1.7–2.5	2.1 (0.2)

**Table 2:** Background nutrients in Coweeta streams.

		NH_4_^+^-N		NO_3_^−^-N	
Stream	Date	Range	Mean (SE)	Range	Mean (SE)
Cunningham	2008	bd – 6.5	bd	bd – 7.0	bd
	2009	5.7–11.1	8.1 (0.8)	bd – 20.4	6.6 (2.4)
	2010	bd – 11.6	6.5 (1.7)	bd - 25.9	12.1 (3.8)
	2011	bd – 15.7	6.6 (1.7)	bd – 13.9	7.1 (1.2)
Hugh White- Lower	2008	bd – 9.6	6.0 (0.8)	15.2–53.1	31.9 (5.1)
	2009	5.9–8.7	7.1 (0.4)	36.9–43.3	39.9 (1.0)
	2010	bd – 19.5	9.2 (2.8)	29.7–65.0	48.5 (5.1)
	2011	bd – 7.5	5.4 (0.7)	17.7–86.9	40.3 (12.8)
Hugh White- Upper	2008	bd – 10.0	7.3 (0.7)	10.8–23.4	15.2 (4.1)
	2009	5.7–7.2	6.9 (0.3)	17.6–23.7	20.4 (1.0)
	2010	bd – 6.7	bd	bd – 30.6	16.3 (3.6)
	2011	bd – 8.7	bd	14.4–57.4	24.4 (6.8)
Mill	2008	bd – 8.9	5.6 (1.0)	bd – 59.3	24.6 (11.0)
	2009	bd – 11.0	8.1 (1.0)	bd – 11.3	7.8 (1.0)
	2010	bd – 9.1	5.2 (1.3)	bd – 11.5	7.8 (1.6)
	2011	bd – 6.1	bd	bd – 13.1	8.3 (2.1)
Reynolds	2008	bd – 20.7	8.0 (2.2)	52.0–99.3	74.8 (5.6)
	2009	bd – 7.3	5.4 (0.6)	6.9–24.8	19.0 (4.1)
	2010	bd – 16.0	7.7 (2.0)	17.6–45.0	33.1 (5.1)
	2011	bd – 18.8	6.2 (3.2)	11.6–29.9	21.5 (3.0)

Background nitrogen (µg L^−1^) in low-order streams at Coweeta Hydrologic Laboratory over the course of this study. Soluble reactive phosphorus was also measured but was always below detection.^1^

1 bd = below detection (5 µg L^−1^).

### Physical Stream Measures

Stream discharge (Q) at all sites was estimated using the sodium chloride slug method [35] prior to measures of metabolism and uptake (described below) each year. Stream wetted widths, depths, and cross sectional areas were also measured during each sampling period.

### Light

Full methods for collecting light intensity and canopy openness data were described previously [13]. In short, light intensity over the study period was collected using HOBO Pendant data loggers (Onset Computer Corp., Bourne, ME, USA) mounted on 1-m tall posts every 10 m along each stream in the riparian zone. Data for relative light intensity (in lux) were recorded every 5 minutes since the beginning of the experiments in 2007. Here, we only report values for July light intensities in order to pair them with our measures of metabolism and nutrient uptake taken during that month each year. Annual patterns in light intensity for these streams have been reported in a previous study [13]. Canopy openness at each site was measured annually prior to leaf-out at permanent stream locations using a digital camera [13].

### Metabolism

We monitored in-stream ecosystem respiration (ER) and GPP every July during the study period using the open-channel, one-station method (e.g. [36], [37]). Multi-probe sondes (Hach-Hydrolab ) were placed in each stream to record dissolved oxygen concentrations (mg L^−1^), oxygen saturation (%), temperature (°C), and specific conductance (µS cm^−1^) every two minutes for 36 hours. Sulfur hexafluoride (SF_6_) injections occurred simultaneously with metabolism measurements and ammonium injections (described below). Replicate water samples were injected into evacuated glass vials to allow for the headspace to accumulate SF_6_.

Headspace air was analyzed on a gas chromatograph (SRI 8610/9300 equipped with an ECD detector, SRI Instruments, Torrance, CA 90503) for relative abundance of SF_6_, and reaeration was estimated using the slope of the log-corrected loss of gas with distance downstream. Data for net ecosystem production (NEP) were calculated by the equation NEP = GPP – ER, where more positive values indicate an autotrophic system and negatives suggest more heterotrophy [38].

### Uptake

We estimated ammonium (NH_4_^+^) uptake using the plateau injection method [39]. In order to not over-fertilize the stream (e.g. [40]), we attempted to raise in-stream NH_4_^+^ concentration by <50 µg L^−1^ over background. A co-injection of ammonium and chloride was added to each site at a known rate with a FMI metering-pump, until specific conductance of the stream (as measured with a YSI-30 handheld conductivity probe, Yellow Springs, OH) reached plateau. Replicate samples of stream water were then filtered through 0.7-µm GFF syringe filters into plastic sample bottles at 6 to 7 stations along the length of the stream reach. Samples were frozen prior to analysis.

Ammonium concentrations were determined using the phenate method [41] with a flow-injection analyzer (Lachat Quickchem 8500, Lachat Corporation, Loveland, CO). Nitrate and chloride were estimated using ion chromatography (Dionex DX500 ion chromatograph, Thermo Fisher Scientific, Inc., Waltham, MA, USA). The log-transformed loss of nutrient over stream length was used to derive an uptake length (S_w_; [42]). S_w_ is the average distance traveled by a nutrient atom as it goes from inorganic to organic form [39]. The uptake length was then used to calculate areal uptake (U) [39]. We chose to use ammonium as our measure of nutrient uptake as it is more readily immobilized than nitrate in Coweeta streams [43], [44], [45]. Additionally, ammonium uptake has been used as a measure of nutrient cycling in numerous studies, contributing to long-term datasets at Coweeta [32], [43], [44].

### Statistical Analyses

Changes in nutrient uptake parameters, GPP, ER, NEP, and July relative light intensities over time were analyzed using Regional Kendall tests for trend, which is a non-parametric test that examines monotonic trends in long-term data [39], [45]. Differences in uptake and metabolism were analyzed separately for year and site using Kruskal-Wallis tests, with post-hoc Wilcoxin multiple comparisons [46]. Non-parametric Pearson correlations were performed between functional parameters and stream physicochemical factors. Computations for the Regional Kendall tests were completed using the software described in [45]. All other analyses were performed on JMP v.9.0 statistical software (SAS Institute, Cary, NC).

## Results

### Light

**Figure 1:**
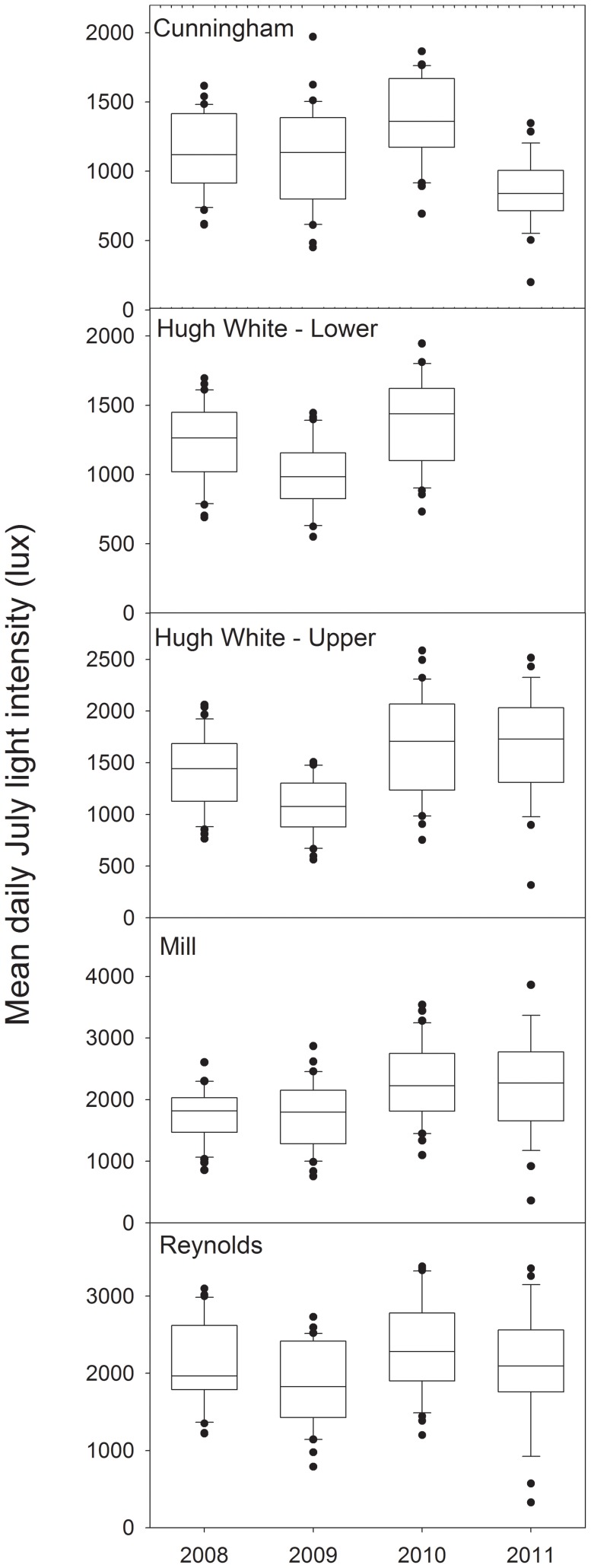
Light penetration in Coweeta streams. Distribution of July light intensities (lux) at the five study sites in Coweeta Hydrologic Laboratory over the course of the study.

Light intensities to streams significantly increased throughout the Coweeta watershed since 2006 [13], with canopy openness also increasing significantly in both sections of Hugh White Creek and Reynolds. In our study, which focused only on July, we see variable light intensities in the streams over time, with only small increases in 2010 (Fig. 1). Even though some sites (e.g. Mill) show an upward trend in light intensity, these changes were not statistically significant (Kendall τ = 0.07, p = 0.90).

### Nutrient uptake

Background levels of ammonium remained low over the course of the study period in all sites (CV = 0.25), while nitrate concentrations were greater and more variable (CV = 0.76, Table 2). Hugh White Creek (both upper and lower sites) and Cunningham had the most consistent nitrate levels during the study. Mill and Reynolds both had peak nitrate during 2008, with much lower concentrations in subsequent years (Table 2).

**Figure 2:**
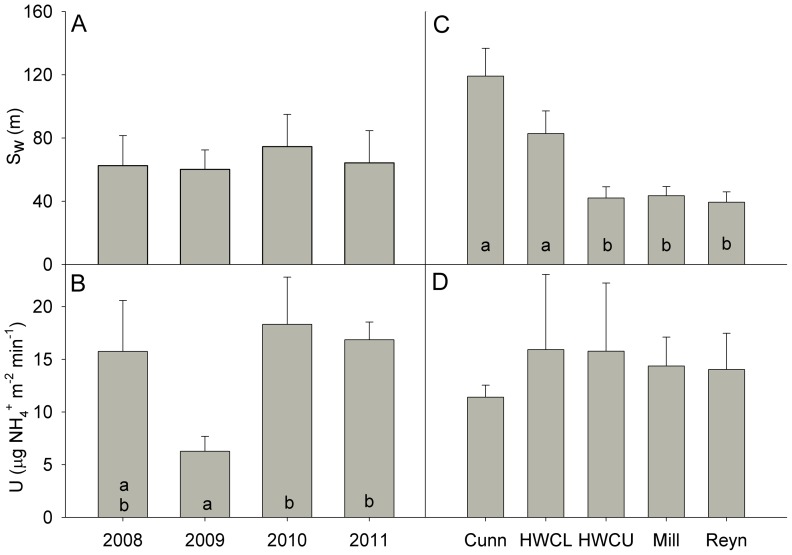
Nutrient uptake parameters in Coweeta streams. Changes in ammonium uptake length (S_w_) and areal uptake (U) for low-order streams in Coweeta Hydrologic Laboratory over the years of the study (A–B) and in each stream (C–D). Error bars represent 1 SE of the mean, and different letters represent significant differences (p<0.05) based on Wilcoxon post-hoc multiple comparison tests.

Both S_w_ and U were variable across sites and years (CV = 0.60). No overall trends in S_w_ (Kendall τ = 0, p = 1.0) or U (Kendall τ = 0.40, p = 0.10) were seen among sites over the course of this study. Uptake was significantly lower only in 2009, but S_w_ was statistically similar throughout the four years of the study (Fig. 2A–B, Table 3). Stream-specific changes in uptake parameters were only seen in Cunningham and Lower Hugh White Creek, both of which had significantly longer uptake lengths than Upper Hugh White Creek, Mill, and Reynolds (Fig. 2D–E).

**Table 3:** Relationships between variables in this study.

Parameter	Light Intensity	Canopy Openness	Temperature	Discharge	Width	Depth	NH_4_-N	NO_3_-N	BOM	S_w_	U	GPP	ER	NEP
S_w_	−0.57*	−0.52*	0.41	0.43	−0.52*	0.49*	−0.07	−0.11	−0.70	-	−0.32	−0.12	0.08	−0.10
U	0.45	0.03	0.15	0.51*	−0.10	0.10	0.09	0.11	0.60	-	-	0.36	0.21	−0.19
GPP	0.33	0.36	−0.20	0.18	0.10	0.13	0.09	0.23	−0.36	-	-	-	0.18	−0.15
ER	0.14	0.15	−0.12	0.04	−0.19	0.25	0.12	−0.04	0.90*	-	-	-	-	−0.99*
NEP	−0.12	−0.13	0.09	−0.07	0.19	−0.27	−0.08	0.06	−0.90*	-	-	-	-	-

Pearson correlation coefficients (ρ) between stream functional parameters and other factors examined in this study. Benthic organic matter (BOM) values from [13] were used for these correlations.^1^

1 Coefficients with (*) indicate significance at p<0.05.

Uptake parameters generally related to physical, as opposed to chemical or metabolic factors (Table 3). S_w_ was negatively related to light (both canopy openness and light intensity) and positively associated with depth, while U showed no relationship with light (Table 3). Uptake was more strongly related to discharge, while S_w_ responded more strongly to stream size (e.g., width and depth; Table 3). The availability of inorganic nitrogen species appeared to not have a significant relationship with any uptake parameter.

### Metabolism

**Figure 3:**
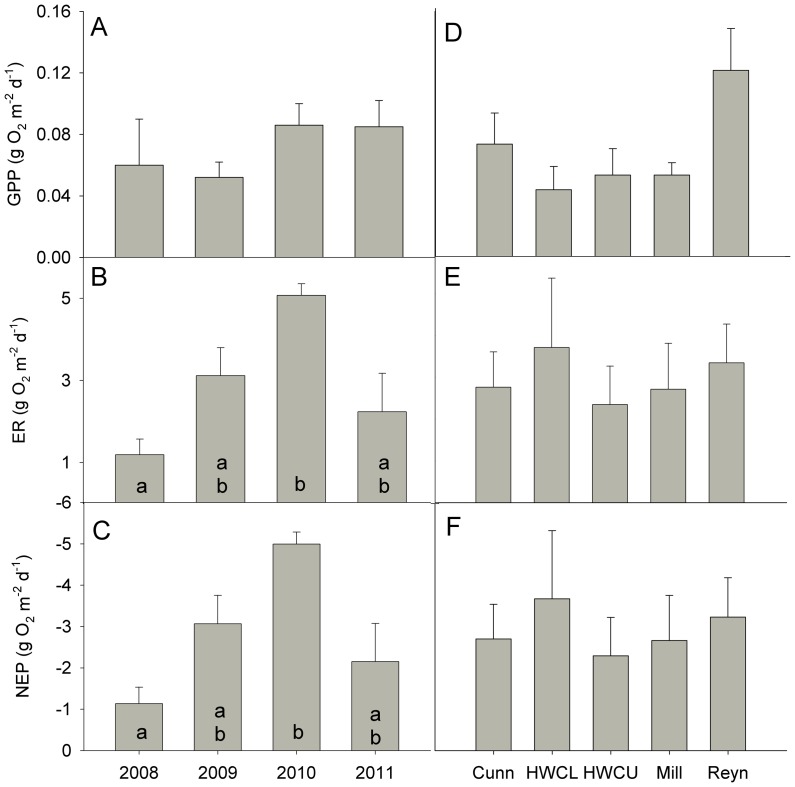
Metabolic parameters in Coweeta streams. Changes in gross primary production (GPP), ecosystem respiration (ER), and net ecosystem production (NEP) for low-order streams in Coweeta Hydrologic Laboratory over the years of the study (A–C) and in each stream (D–F). Error bars represent 1 SE of the mean, and different letters represent significant differences (p<0.05) based on Wilcoxon post-hoc multiple comparison tests.

Metabolic parameters were quite variable over stream locations and across years (CV = 0.60 for both GPP and ER). Levels of GPP were very low (Fig. 3A,D), ranging from 0.01 to 0.18 g O_2_ m^−2^ d^−1^ in Upper Hugh White and Reynolds Creek, respectively. ER ranged from a low of 0.4 g O_2_ m^−2^ d^−1^ in Lower Hugh White Creek in 2008 to a high of 5.4 g O_2_ m^−2^ d^−1^ in Mill Branch in 2010. Negative values of NEP indicated that all streams were net heterotrophic at the times of our measurements (Fig. 3).

As with uptake, no overall trends in metabolism were seen across sites over the course of this study: GPP (Kendall τ = 0.27, p = 0.27), ER (Kendall τ = 0.27, p = 0.29), NEP (Kendall τ = −0.27, p = 0.29). Strong differences in stream metabolism were seen in 2008 and 2010 (Fig. 3A–C), with NEP significantly lower in 2010 compared to 2008 (Fig. 3C). This effect was mostly driven by significant differences in respiration (Fig. 3B, Table 3), as GPP was not significantly different among years. No significant differences in metabolic parameters were found among sites over the course of the experiment (Fig. 3D–F).

There were no significant relationships between stream physicochemical variables and metabolic parameters (Table 3). Uptake parameters also had no significant associations with metabolism (Table 3). There appears to be a suggestive response of ecosystem respiration and NEP to changes in benthic organic matter availability (Table 3), although these relationships were only based on data for 2008.

## Discussion

### Primary Production

Contrary to our predictions, there has been no appreciable change to in-stream GPP, even with basin-wide increases in light availability to streams. Historically, Coweeta streams had low nutrient concentrations [47], [48] and have been shown to be nutrient-limited [26]. Our data demonstrate that this condition has not changed over the course of our study (Table 2), in spite of documented losses of nitrogen from terrestrial watersheds affected by HWA [5], [23]. Even if light becomes more available to stream ecosystems, autotrophic production is dependent on the availability of inorganic nutrients, especially N and P [49], [50], [51]. Further, nutrient limitation of primary production may only become apparent under conditions where light is more abundant [52].

Although we were unable to demonstrate trends in uptake over time, the generally short S_w_ and low overall uptake are consistent with previous studies [44], [53], [54], [55] suggesting that streams of this region are nutrient limited [26]. Significantly shorter S_w_, coupled with similar levels of uptake in Upper Hugh White Creek, Mill, and Reynolds potentially indicate increases in N-immobilization due to hemlock mortality [18]. Even though hemlock detritus is a relatively low-quality resource, a previous study in Coweeta [53] suggested that the woody input from dying trees may provide a greater surface area for microbial colonization and nutrient immobilization as suggested by our data.

Light limitation of autotrophic production in these headwater streams may not be alleviated by the canopy loss due to HWA infestation. Previous work in Coweeta demonstrated significant annual increases in light intensity across the basin over the 4 years of their study [13]. Further, light limitation has been demonstrated in heavily shaded streams in Tennessee during the summer months after manual removal of riparian vegetation, where there was a significant increase in in-stream primary production [55]. Even so, this short-term experiment did not demonstrate any compositional changes to riparian plant communities in White Oak Creek, Tennessee. Therefore, shading (not species composition) was a more significant contributor to rates of in-stream primary production in this low order forested stream.

The increase in light that was evident from annual means [13] was not observed in July. Instead, July light has been relatively unaffected over the 4 years of this study (Figure 1). The greatest differences in canopy openness and light infiltration into the streams occur during the winter and early spring months of the year [13], but we do not have annual estimates for uptake and metabolism to pair with these annual patterns in light. Thus, we were only able to address relationships between light, metabolism, and nutrient uptake during the times when we have data for each of those factors (i.e. July).

While canopy opening due to HWA infestation does increase light availability, growth of other understory and riparian species may be stimulated. Significant, negative relationships between rhododendron and hemlock have been demonstrated previously [13], suggesting a possible suppression of rhododendron due to previously heavy hemlock cover. With the loss of hemlock canopy in watersheds affected by HWA, rhododendron appears to be re-establishing close to streams [3], [7], [56]. The lack of July trends in light data here also suggests that riparian re-growth of rhododendron is providing shading to the streams that was previously due to hemlock canopy, thus continuing to suppress primary production in our system.

### Metabolic changes and respiration

Changes in GPP were not associated with metabolic change, while more significant changes to NEP did occur due to ER (Fig. 3, Table 3). Although forested headwater systems such as these are typically heterotrophic (e.g. [57]), there appeared to be movement towards even greater heterotrophy from 2008 to 2010 (Fig. 3). Although we did not have complete BOM data throughout our study, the significant relationship between BOM, ER, and NEP does suggest an even greater role for organic matter in controlling ecosystem metabolism (Table 3).

Large amounts of wood have been measured in these streams [13], which is a resource that may remain in the streams for decades [53], [58], [59]. Reynolds Branch, which had a large contribution of hemlock to overall riparian basal area [13] may be indicative of how streams in this region may respond to further loss of hemlocks. Small shifts in respiration (Fig. 3), possibly due to greater litter fall in Reynolds, combined with relatively short S_w_ (Fig. 2) may indicate a greater heterotrophic nutrient demand (e.g. [31], [60]) relative to Cunningham and HWC.

### System-wide effects and future work

In Coweeta, the immediate effects of infestation by HWA (i.e. mass infestation and killing of hemlocks) may be characteristic of the “acute” stage of invasion [61]. As such, systems undergoing an active disturbance will not show immediate responses due to transitional stages that will occur as ecosystems move between stable states and nutrient retentive ability [56], [62]. We were unable to fully address changes to nutrient dynamics during times of peak light inputs into Coweeta streams, which occur in April [13]. A more thorough, annual assessment of nutrient uptake would be valuable in the future. Our initial predictions of changes to primary production, respiration, and nutrient uptake due to overstory canopy loss may be better addressed over annual time scales.

Future increases in hemlock detritus to streams will also increase standing stocks of organic matter due to the recalcitrance of needles [62] and generally slow breakdown of wood [20], [53]. Further measures of hemlock inputs and subsequent processing of particulates is needed. Additionally, woody inputs may ultimately change the retentive nature of streams [60], [63], thus affecting function in ways yet to be determined (e.g. [63], [64], [65]). These “chronic” effects of HWA invasion, where inputs from dead or dying hemlocks may lead to subsequent alterations of ecosystem structure and function, will take time to manifest in our system [61].

## Conclusions

Even though large, significant changes to metabolism and nutrient uptake were not seen in our study, the inherent variability in the data was even more important. This study, and most others relating to HWA effects, occurred during an active infestation, with the variability most likely indicating that stream ecosystems in Coweeta are transitioning to new steady states. In-stream primary production did not increase over the course of the study, partially due to the consistent shading of streams during July by broad-leaved species. Net ecosystem production changes were mostly driven by increased respiration, likely related to changes in benthic organic matter. On the other hand, nutrient uptake showed no consistent patterns over time. The full extent of the impacts of HWA-mediated hemlock loss on stream ecosystems may not be known for decades.
